# Socialization in Commercial Breeding Kennels: The Use of Novel Stimuli to Measure Social and Non-Social Fear in Dogs

**DOI:** 10.3390/ani11030890

**Published:** 2021-03-20

**Authors:** Margaret Pritchett, Shanis Barnard, Candace Croney

**Affiliations:** Department of Comparative Pathobiology, Purdue University, 725 Harrison Street, West Lafayette, IN 47906, USA; pritche3@purdue.edu (M.P.); ccroney@purdue.edu (C.C.)

**Keywords:** behavioral assessment, *Canis familiaris*, commercial dog breeding, fear, welfare

## Abstract

**Simple Summary:**

Owner-based reports of dogs presumed to come from commercial breeding kennels (CBKs) suggest high levels of fear in this population. Fear in kenneled dogs is a serious behavioral welfare concern as it may lead to both acute and chronic stress. Novel social and non-social stimuli have been shown to elicit behaviors associated with fear in animals. New knowledge on the levels of fear in dogs from CBKs could be used to further refine protocols intended for assessment of welfare in CBKs and to improve breeders’ management practices. The aim of this study, therefore, was to evaluate how dogs from CBKs reacted to social (i.e., a person approaching) and non-social (i.e., a traffic cone and a dog statue) stimuli, and to perform a preliminary evaluation of how these responses might be used as indicators of dogs’ overall socialization levels in kennels. Results revealed that dogs had primarily mildly fearful responses to the stimuli presented. These findings are encouraging as extreme fearful reactions were rarely recorded. Nevertheless, there is a clear margin for commercial breeders to improve the socialization protocols in their kennels to better incorporate both social and non-social stimuli.

**Abstract:**

Understanding the behavioral welfare of dogs in commercial breeding kennels (CBKs) is important for improving breeders’ management practices as well as dog welfare. In the current study, breeding dogs from CBKs were exposed to novel stimuli to evaluate their behavioral responses, with emphasis on indicators of fear. Subjects were presented with a standard stranger-approach test, a traffic cone, and a realistic dog statue. Sixty dogs were exposed to the three stimuli and behavioral responses were scored using an ethogram developed for this study. Dogs spent significantly more time investigating the environment, staying further away from the stimulus, and they took longer to approach and investigate when presented with the cone than with the dog statue or stranger (*p* < 0.01). These findings suggest that the cone elicited more fear-related behaviors than the dog statue and stranger. Given these results, in addition to socializing their dogs to unfamiliar people and other dogs within their kennels, commercial breeders should be encouraged to increase the exposure of their dogs to more diverse novel stimuli to reduce non-social fear and support the welfare of dogs while they reside in the kennel and when they transition to new homes.

## 1. Introduction

Until recently, studies focusing on the behavior and welfare of dogs from commercial breeding kennels (CBKs) were scant [1]. While research on the behavior of these dogs is still fairly limited, basic knowledge from investigations of similarly confined dog populations, such as those kept in shelters or laboratories, may have implications on the lives of dogs from CBKs [2,3,4]. For example, studies have illustrated the importance of housing quality, predictability of the social environment and frequency and quality of human–animal interactions for animal welfare and longevity [5,6,7,8]. Previous research on the welfare of dogs presumably from CBKs conducted using data originating from dog owner reports [9] has lacked empirical evidence from direct observations. However, with advances in the development of dog welfare assessment tools and increased direct access to commercial dog breeding premises [10,11,12,13], researchers are beginning to better understand the behavior and health status of this population and how to assess their overall welfare in a more holistic way. It is important for researchers, breeding facility operators, and inspectors to have access to highly functional, validated, and easy to use tools designed to measure canine welfare in the field. For example, the development of the Field Instantaneous Dog Observation (FIDO) tool, which offers a means to assess the physical and behavioral welfare states of dogs in CBKs, including their physical and behavioral health, has played an important role in the move toward the collection of direct observational data from this population [10].

An animal’s welfare can be greatly impacted by high levels of fear or a predominantly fearful emotional state, which may lead to both acute and chronic stress [14,15]. Dogs experiencing acute stress are more likely to exhibit submissive and/or fear-related behaviors, such as paw-lifting and lowered postures, whereas prolonged chronic stress has been shown to induce behavioral stereotypes [5]. Understanding the levels of fear in CBK populations, and how fear may impact dogs’ quality of life after transitioning out of the kennel, is a necessary line of inquiry to ensure their welfare. At the end of their breeding careers, eligible dogs from CBKs may be rehomed as pets. For inadequately socialized dogs, experiencing high levels of social and non-social fear could seriously impact their ability to transition smoothly to their new homes. This is critical because dogs that present major behavioral problems once rehomed may be at high risk of abandonment, surrender to a shelter or euthanasia [6]. Stella and colleagues [11] suggest that the assumption that all retired dogs from CBK are equally good candidates for rehoming might significantly compromise the safety and welfare of those more fearful individuals. The authors advised that, for these dogs, there should be a greater degree of socialization in place to help prepare them for such transitions.

Both social and non-social stimuli have been shown to elicit behaviors associated with the flight or fight response in dogs, with social stimuli provoking a higher frequency of fear-related behaviors [15]. However, to date, there are no published studies that quantify the levels of fear in dogs from CBKs based on their behavioral reactions to both social and non-social stimuli. Such information could potentially be used to further refine behavioral assessment protocols intended for assessment of welfare in CBKs and to maximize rehoming success after retirement. Additionally, better understanding of social and non-social fear responses in this population of dogs could help to inform standards of care and management practices for dogs raised in CBKs. The aim of this study, therefore, was to evaluate how dogs from CBKs reacted to social (i.e., an unfamiliar person approaching) and non-social (i.e., a plastic traffic cone and a dog statue) stimuli, and to gauge the degree to which behavioral responses to exposure to these stimuli might be used as indicators of the adequacy of socialization practices used in CBKs.

## 2. Materials and Methods

### 2.1. Ethics Statement

The procedures described were reviewed and approved by the Purdue University Institutional Animal Care and Use Committee (PACUC 1809001796), and permission to visit the kennels and record the videos was granted by the owners prior to the commencement of the study.

### 2.2. Subjects

The subjects for the current study (*n* = 60) were randomly selected from a pool of 447 dogs from 26 CBKs, located across the Midwestern United States. These subjects were part of a larger data collection effort for another study (ongoing) which aimed to investigate management and welfare risk factors affecting rehoming outcomes in retiring dogs from CBKs. Within that larger study, dogs were assessed using a refined version of the FIDO tool [10] and categorized as “red”, “yellow” or “green” (RYG). As reported by Bauer et al. [10], the assessment was based on an unfamiliar person approaching the front of the pen, maintaining a sideways orientation, and scoring the immediate behavioral reaction of the dog. A dog was scored “red” if it showed signs of fear and/or stereotypic behavior; “green” if it showed affiliative approach, solicited attention or was undisturbed by the presence of the approaching tester; or “yellow” if it showed an ambivalent approach/avoidance behavior or could not clearly be scored “red” or “green”. Tests were videotaped for later analysis. The subsample of 60 dogs was selected from the main data spreadsheet (Excel) using a random number generator (google.com). The subsample included 36 dogs scored as “green”, 11 scored as “yellow”, and 13 scored as “red”. There were 15 males and 45 females. Furthermore, this population consisted of a variety of 26 purebreds and designer crossbreeds (Table 1).

The average age of the dogs was 3.4 years (range = 1 to 7, SD = ±1.42). Physical health data collected using the FIDO tool [10] showed overall good health conditions for all dogs: no coughing, sneezing, lameness, nasal discharge or wounds were observed and body condition was normal to stout. Tear staining or ocular discharge was observed in half of the dogs. Dogs had clean coats: only 10/60 dogs had mild spotting (i.e., less than 25% of the body wet or with debris).

The sampled dogs were from 20 different commercial breeding facilities, representing different housing systems, varied flooring types/materials (e.g., concrete, tenderfoot), indoor pen sizes (ranging from 0.7 to 4.5 m^2^, mean = 2.3 m^2^, SD = 1.2 m^2^), varied access to the outdoors (e.g., indoor only or with free indoor/outdoor access), and access to separate exercise areas (e.g., additional daily or weekly access to separate outdoor exercise yards). The breeders enrolled in the original study volunteered their participation, and their kennels all exceeded minimum space and exercise requirements for dogs mandated by U.S. federal law [16].

### 2.3. Procedure

The three stimulus–response tests used in this study were conducted by two female researchers. During the test, dogs were confined into the indoor portions of their home pens (i.e., they had no access to the outdoor) to ensure availability for scoring. As the indoor pen size varied in dimension as previously described, and because dogs were free to move within the pen space (i.e., the tested dog was not positioned at a specific starting location to avoid additional handling stress), the distance between the stimuli and the dog at the start of the test varied. The first stimulus–response test conducted was a three-step stranger approach in which the tester: (1) opened the pen door with a sideways orientation and without making direct eye contact with the focal dog, (2) offered a treat to the dog directly from her hand, always maintaining a sideways orientation, and (3) offered a second treat from one hand while reaching to gently touch the dog with the other hand. Finally, the tester stepped back from the pen and closed the gate (adapted from [11]). The second response test consisted of placing a plastic orange traffic cone into the pen with the dog and locking the pen gate. The cone was left in the pen and the dog was allowed to explore it for 30 sec before the tester removed it. The final stimulus–response test included the placement of a realistic dog statue (Boston terrier figurine, 40 cm height) in the pen with the dog. Again, the dog was allowed 30 sec to investigate the object before it was removed by the researcher. The objects were thoroughly disinfected between kennels. If the tested dog licked, chewed or eliminated on the objects, then these were cleaned and let to air out before moving to the next dog. It is important to note that this set of tests was selected in order to elicit a variety of responses, from interest to mild fear, without provoking extreme reactions of avoidance and aggression that could have harmed the animals.

Each of these tests was video recorded using a digital video camera (Sony Handycam HDR-CX405) mounted on a tripod. Videos (3/dog = 180 video clips) were subsequently analyzed using the behavioral scoring software BORIS (Version 7.8.2). The dogs’ behaviors during each test were analyzed using an ethogram based on the available literature on fear and stress in dogs [5,14,15,17]. The behavioral variables were then grouped into eight main categories: fear, stress, aggression, stereotypic behaviors, activity, vocalization, and non-fearful investigation. The full ethogram used to code the videos is provided in Table 2. The ethogram was pilot tested with a small additional subset of videos (*n* = 9) not included in the analysis.

### 2.4. Analysis

All statistical analysis was performed using SPSS (IBM, Version 26). To assess intra-rater reliability, nine subjects were randomly selected (15% of the sample), and all three reaction tests for each subject were re-analyzed from video by the same observer two months after initial scoring. Levels of agreement were determined using Intraclass Correlation Coefficients and interpreted as follows: ICC < 0.50, poor agreement; 0.50–0.75, moderate agreement; 0.75–0.90, good agreement; >0.90, excellent agreement [18].

For the following analysis, each dog acted as its own control. Wilcoxon sign-ranked tests were conducted to compare time spent at the front of the pen versus the back of the pen. This was used to gauge the general willingness of each dog to engage with or avoid the stimulus (i.e., stranger, traffic cone, or dog statue) positioned at the front of the pen. Kruskal–Wallis analysis of variance tests were conducted to compare the behavior of dogs across the three stimuli. For significant results, Wilcoxon tests were used for post hoc pair comparisons, applying Bonferroni correction (*p* < 0.016).

Additional analyses focused on sex, age and pen size to examine differences between behavioral variables of interest. For these analyses, behaviors were considered independently of the stimuli (i.e., same behaviors were summed across stimuli). The breeds of dogs were also recorded; however, there were not enough subjects in each breed to have the requisite power to perform breed comparisons. A Wilcoxon sign-ranked test was used to compare behavioral differences of male versus female dogs. A simple logistic regression was calculated to indicate any possible associations between the behavioral variables and the dogs’ ages and pen sizes.

Descriptive analysis was used to characterize behaviors that were only seen in the stranger approach test: specifically, the “affiliative approach to tester”, “contact/no contact”, and “takes treat” data. Finally, cross tabulations were created to explore whether showing higher levels of sociability towards unfamiliar people was associated with fewer signs of fear towards non-social stimuli. This was performed by comparing each dog’s “red”, “yellow”, or “green” classification from the behavioral portion of the previously conducted FIDO testing with their response to the cone and dog statue.

## 3. Results

Due to their very low occurrence, some behaviors were excluded from the analysis, including “growl”, “whine”, “body shake”, “yawning”, and “shivering”. Similarly, “biting” and “teeth baring” were never seen, and “pacing” and “circling” were only noted two and three times, respectively, out of the 180 observations. Behaviors associated with more intense fear reactions (i.e., “escaping” and “retreat”) were observed in two dogs (less than 2% of the sample) during the stranger approach test, in three dogs (less than 5% of the sample) during the response to the dog statue test and in four dogs (less than 7% of the sample) during the response to cone test. Thus, these behaviors were also excluded from the analysis. Behaviors not included in the analysis are indicated in Table 2. Other behaviors (i.e., “bark”, “paw-lifting”, and “lip-licking”) were combined into a larger “stress-related” category for analysis. Additionally, postures (i.e., “lowered posture”, “neutral posture”, and “rigid posture”), which were included as modifiers (e.g., attributes of behaviors), were collapsed into single independent variables (i.e., independently of the behavior they were associated with) for analysis.

Intra-rater reliability analysis confirmed a good agreement (0.76), on average, across all behavioral variables. An excellent agreement was observed for 58.8% of the variables analyzed (10/17), while the remaining variables showed a moderate to good agreement (0.56–0.80, Table 3).

When analyzing the time spent in the front or back portions of the pens during each stimulus–response test, a Wilcoxon test revealed that dogs spent significantly more time in the back of their pens, compared to the front, when introduced to the cone (Z = −2.113; *p* = 0.035). No significant difference was found for time spent in each portion of the pen when dogs were introduced to the other two stimuli. However, on average, dogs spent more time at the back of the pen (i.e., further away from the stimulus) with the dog statue (mean: back = 5.44 s; front = 3.16 s) and more time at the front of the pen (i.e., closer to the stimulus) during the stranger interaction (mean: back = 5.45 s; front = 7.14 s).

**Figure 1:**
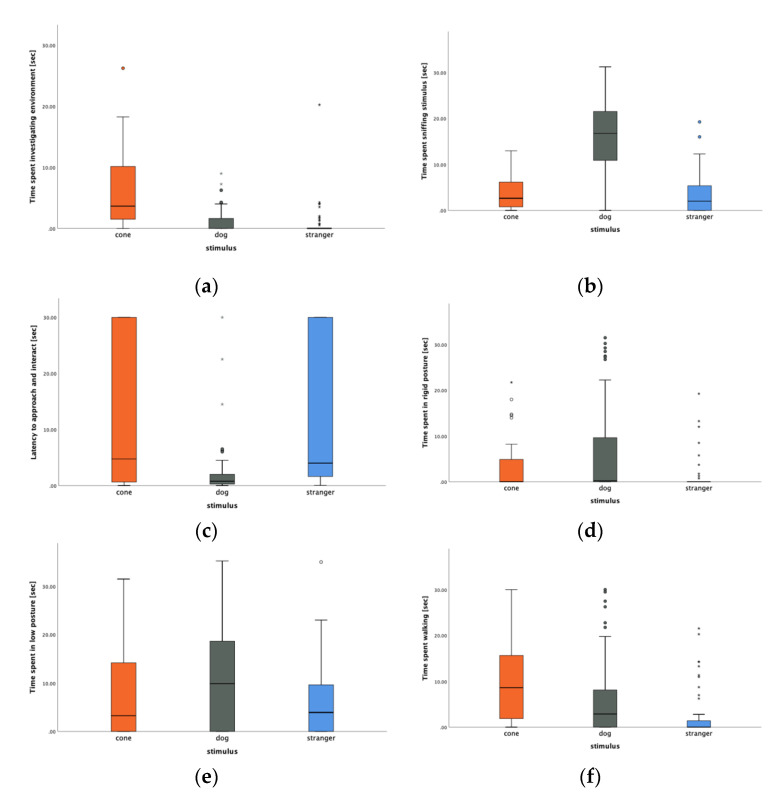
Boxplot representing the time spent by dogs (**a**) investigating the environment, (**b**) sniffing, (**c**) latency to approach and interact, (**d**) in a rigid posture, (**e**) in a low posture, and (**f**) walking, when presented with three different stimuli: a traffic cone (cone), a dog statue (dog), and an unfamiliar person approaching (stranger). Values represented are: medians (bar within the box), upper and lower quartiles (borders of box), lowest and highest cases within 1.5 times the IQR (bottom and top whiskers) and outliers (circles and asterisks).

Statistically significant differences in the dogs’ responses to the three stimuli for the following behaviors were demonstrated by the Kruskal–Wallis test: “investigating the environment” (χ^2^ = 59.8, *p* < 0.0001), “sniffing the stimulus” (χ^2^ = 75.8, *p* < 0.0001), “latency to approach and interact with the stimulus” (χ^2^ = 53.8, *p* < 0.0001), “low” posture (χ^2^ = 9.84, *p* = 0.007), “rigid” postures (χ^2^ = 15.3, *p* < 0.0001), and “walking” behaviors (χ^2^ = 28.5, *p* < 0.0001). Post hoc comparisons revealed that the subjects spent significantly more time “investigating the environment” when presented with the cone compared to the dog statue or the stranger (*p* = 0.0001, Figure 1a). Additionally, dogs spent significantly more time engaged in “sniffing” behavior and had a shorter latency to approach and interact when presented with the dog statue compared to the cone or the stranger (*p* = 0.0001 for both behaviors, Figure 1b,c). Pairwise comparisons also indicated that dogs spent more time displaying rigid postures when presented with the dog statue than when presented with the cone and the stranger (*p* = 0.010 and *p* = 0.016, respectively, Figure 1d). They also spent more time in lowered postures when presented with the dog statue compared to the stranger (*p* = 0.001, Figure 1e) but not with the cone. Finally, dogs spent less time performing “walking” behavior during the stranger approach test than they did when presented with either the cone or dog statue (*p* = 0.0001, Figure 1f).

Further analysis revealed no significant differences in behavioral responses for all stimuli between male and female subjects. Pen size was found to influence a dog’s position within the pen: dogs in larger pens (i.e., more square meters of surface) spent less time in the back of pen compared to dogs in smaller pens (*t* = −2.08, *p* = 0.042). All other associations were non-significant.

For the behaviors observed only during the stranger approach test, dogs spent on average 0.68 sec in affiliative behavior (2.27% of total observation time, range: 0 to 9.5 sec; SD = ±1.68). During the stranger approach test, the tester attempted to give the subject a treat from their hand as well as to touch the subject with their hand. In this interaction, the tester was able to give the dog a treat directly 30 times (50% of subjects) and to make contact 25 times (41.67% of subjects).

Cross tabulations revealed how dogs’ reactions to the cone (Table 4) and the dog statue (Table 5) were associated with their levels of sociability towards people, based on the Red–Yellow–Green (RYG) assessment.

## 4. Discussion

The goal of this study was to evaluate the effects of different stimuli in eliciting social and non-social fear in a population of dogs from CBKs and to perform a preliminary evaluation of how these responses might be used as indicators of dogs’ overall socialization levels in kennels. In this study, we recorded intense fear responses, such as freeze, escape attempt, or aggression, elicited by the three selected stimuli in very few animals (maximum 4/60 dogs). Instead, milder signs of fearful reactions were observed, including preferences for the back portions of their pens away from test objects, actively avoiding and increasing the distance from the stimuli, taking longer to approach and interact with, or complete failure to come into contact with the stimuli. The absence of extreme or even relatively strong fear responses may indicate that these dogs were somewhat able to cope with the challenges presented and, in turn, suggests that even though there is ample space for improvement, some level of effective socialization may have already taken place at these kennels. It is important to note that these results may also have been mitigated by the test design, which was not intended to provoke intense fear responses. For completeness, it should be mentioned that the response recorded toward these stimuli may be exclusive to this study population. Due to the lack of an external control group, it is not possible to determine if the type of response these stimuli would elicit in other populations (such as pet and shelter dogs) differs from that of dogs from CBKs. This goes beyond the scope of this paper as our aim was not to compare the prevalence of fear behavior in CBKs to an expected outcome; however, it is something to keep in mind in future applications of this test. Finally, the dogs used in this study came from breeders who volunteered to participate, so results may not be reflective of all U.S. commercial breeding kennels.

From our analysis, the orange traffic cone, a non-social stimulus, triggered significantly higher rates of fear-related behaviors compared to social (or presumed social) stimuli, such as the stranger and the dog statue. The dogs’ fear when introduced to the cone was expressed through more time spent in the back portion of the pen away from the cone (which was placed in the front portion of the pen). This position in the pen was not observed as frequently when the dogs were presented with the other two stimuli. It should be considered that not only was the back portion of the pen further away from the stimulus, but it typically gave dogs access to outdoor runs. It is therefore likely that dogs showing a preference for the backs of their pens may have been looking to escape even though they were not actively scratching at the doors. A second indicator of the uneasiness provoked by the cone was that dogs spent more time investigating the environment than the cone. While dogs may spend a considerable amount of time investigating new environments, the only new environmental element presented during this test was the cone itself. This extended activity may, therefore, have been a type of redirected investigative or coping behavior [19], suggesting that the cone was creating an emotional conflict and leading dogs to perform alternative investigative behaviors. As the cone represented a novel, motionless object to which this population of dogs had never been exposed, these findings may suggest that the dogs in our sample may not have been adequately exposed to enough novel objects during their lives in the kennels to have been unafraid of the traffic cone. This finding has welfare implications as dogs that struggle to cope when presented with a motionless object, such as a plastic cone, are also likely to struggle when exposed to an unfamiliar household once re-homed, thus potentially resulting in a stressful transition [11]. It could be argued that the presentation of a novel object is, by design, fear-inducing and that any dog may react fearfully to such an exposure, even if well-socialized, so conclusions about lack of socialization should be drawn with caution. The individual variability recorded (as shown by the boxplots in Figure 1) is an indicator that some dogs were not intimidated by the cone or were able to overcome an initial fear and engage with the object. Whether this variability was due to genetic, environmental, or experiential factors is not possible to say at this point. Therefore, it is important that future intervention studies investigate if the implementation of specific socialization protocols does indeed reduce the overall level of fear towards novel objects in CBK populations or if other factors play a major role.

The dog statue elicited a different set of responses from the cone and stranger. Dogs approached the statue significantly faster than they approached the other stimuli. Given that the statue was a realistic reproduction of a life-sized conspecific, it is likely that the dogs’ first reactions were similar to those experienced during a social encounter with a conspecific, i.e., social investigation was the immediate response triggered. Subjects spent more time “sniffing” the dog statue when compared to the other two stimuli. Furthermore, dogs that explored the statue were more often observed in either a “lowered” or a “rigid” posture than those that explored the other two stimuli. Such rigid and lowered postures are typical in agonistic interactions, suggesting that dogs may have initially perceived the statue as an unfamiliar conspecific. On the other hand, a “lowered” posture could indicate either a submissive affiliative behavior, an attempt to solicit social interaction in a non-threatening manner, or maybe a state of fear. Unfortunately, the nature of our study did not enable classification of the emotional states of the dogs during such interactions. However, it should be considered that, while some dogs reacted in an affiliative manner during the first half of the test, they may have switched to fear behavior upon failure to elicit an appropriate social response from the statue or after realizing that this stimulus was actually an inanimate object, like the cone. This is in line with previous studies using fake dogs as proxies for unfamiliar dogs in dog–dog interactions. Barnard et al. [20] found that their subjects responded with the same general behaviors when presented with a fake dog reproduction as with a living dog. Given that the subjects seemed to react (at least initially) to the statue as if it was another dog, it is possible that this stimulus could reflect the response of the tested dog to another unfamiliar dog after being rehomed. The greater amount of exploration and lower initial fear toward the dog statue suggests that the dogs in this sub-sample were generally socialized to other dogs. Indeed, common management practices used by the participating breeders included allowing the dogs into outdoor exercise yards in groups and flexible group composition when socially housing them. Overall, it is interesting that the dogs seemed to respond to the dog statue more as a conspecific than as a non-social object. The use of artificial conspecifics has many advantages, allowing performance of social behavior and cognition experiments in a more controlled and repeatable manner compared to living animals [21,22,23]. The use of artificial dogs during behavioral assessments in homes and shelters has gained attention as it permits testing of dogs safely and in a standardized way, although its validity is still controversial [20,24,25,26]. Future studies should further investigate how dogs perceive social and non-social stimuli and what elements of these stimuli might elicit fear responses. This could potentially impact the design and conduct of future behavioral and cognitive tests, as well as socialization practices in breeding kennels.

The stranger approach test elicited the lowest level of “walking” behavior compared to the other stimuli. In addition, when the stranger was present, dogs spent more time at the front of the pen (i.e., in relatively close proximity to her) and they spent less time moving away from the stranger compared to the cone stimulus. This suggests that the dogs were less fearful of the stranger than the cone. However, it is interesting to note that in the stranger test, the “latency to approach and interact with the stimulus” was similar to that of the cone and was significantly higher than that of the dog statue. This could indicate that the stranger elicited a greater level of fear or a greater level of conflict behavior compared to the dog statue. This finding is further supported by the reduced amount of time dogs spent sniffing the stranger compared to the dog statue. Whether the level of fear induced by the stranger was due to insufficient socialization of these dogs to unfamiliar people or whether it was due to the unfamiliarity of the test situation and the movement of the tester while performing the test is unclear. During the stranger approach tests, the tester was only able to hand-feed a treat to 50% of the dogs, which indicates that many of the subjects were not comfortable enough in the presence of a novel person to approach and take a treat. Additionally, the tester was only able to touch 25 of the 60 dogs, suggesting that the majority were not comfortable with a stranger slowly reaching out and attempting to touch them. Although these dogs were handled daily by their caretakers, it is possible that many were not sufficiently exposed to or handled by unfamiliar people often enough for them to generalize their social responses to new people. In a pilot study carried out by this group (unpublished), it was observed that some dogs reacted fearfully to strangers, even though they were highly social toward their own caretakers. For successful rehoming outcomes, it is critical that dogs are able to generalize positive perceptions of their interactions with their caretakers to new people to whom they are exposed to avoid being chronically distressed. The implementation of effective socialization protocols in CBKs may, therefore, help to achieve this goal. Currently, our research team is investigating if brief daily caretaker interaction may have an effect on the dogs’ behavior, not only toward the caretaker but also, by generalization, toward unfamiliar people. If successful, this could be an easy-to-implement protocol with great beneficial impact.

The cross-tabulation analysis allowed for comparison of a subject’s behavior during the cone and dog statue reaction test and their stranger approach RYG classification. When presented with the cone, dogs categorized as “red” showed greater average durations for fear-related behaviors, such as gaze avoidance and lowered postures than dogs scored as “yellow” or “green”, as well as longer latencies to approach and interact with the stimulus. In contrast, dogs in the “green” category spent a greater average duration performing behaviors such as play, sniffing, and neutral posture than did dogs in other categories. Collectively, these findings support the idea that “green” dogs had lower levels of fear when exposed to the cone stimulus than the dogs in the “red” category. Similarly, when exposed to the dog statue, dogs in the “red” category, on average, spent more time performing gaze avoidance and maintaining a low posture, and demonstrated a higher increased latency to approach and interact with the stimulus than dogs categorized as “green” or “yellow”. When presented with the dog statue, dogs in the “green” category exhibited play behavior, which was completely absent in the dogs scored as “red” or “yellow”. This again suggests that “red” and “yellow” dogs had greater levels of fear in the presence of the dog statue than dogs in the “green” category. It should also be noted that all categories had a high average duration for “sniffing” behavior when presented with the dog statue. This suggests that most dogs were highly interested in the stimulus, regardless of their RYG classification. This observation may have practical applications: a high prevalence of “red” dogs in the kennel may indicate higher likelihood of generalized social and non-social fear in a population. If future experiments confirm this association, this very brief stranger approach test may be used as an easy initial screening tool before performing more comprehensive behavioral assessments.

Although high levels of fear may be a consequence of poor or absent socialization practices in the kennels, it is important to note that a range of different factors may play a role in shaping fear responses. These include genetics, physical health, handling and breeding practices, exercise, conspecific interactions, parity, breed and more [6,27,28,29,30]. In this study, no significant differences were found between the behavioral reactions of males and females to any of the three stimuli. The effect of breed could not be discerned, because breed could not be included in the analysis. However, there is evidence from previous work that breed may have a significant effect on fear levels. More than 50 breeds have been determined to show potentially heritable fear/anxiety [29]. Additionally, Morrow et al. [30] found significant differences in the percentage of subjects demonstrating fear-related avoidance behaviors and the age of onset of these behaviors between three various breeds. Future work building upon the current study should, therefore, include breed as a potential risk factor for fearful temperament. Finally, there was a significant association indicating that dogs in larger pens spent less time in the back portion of the pen than dogs housed in progressively smaller pens. It is likely that dogs in larger spaces could be closer to the front of the pen and still maintain a comfortable distance from the object (placed at the front of the pen). In contrast, dogs in small pens had to retreat to the far end of their pens to create distance from the stimulus presented. There may also have been a confound in that small breeds typically are housed in smaller pens. Hence, future studies should account for both space allocations and breed factors to better understand the effects and impacts of larger pen spaces on the levels of fear expressed and dogs’ coping strategies when faced with a new, challenging situation. This line of research could have an impact on space recommendations for other populations of kenneled dogs such as those maintained in working, shelter and laboratory environments.

A systematic data collection and risk analysis that combines animal, resource, and management metrics could help move toward a reliable quantitative assessment of fear and identify key areas for improvement for dogs in CBKs.

## 5. Conclusions

Findings from this study revealed that the cone stimulus elicited the greatest levels of behaviors indicative of fear than either the dog statue or stranger. It is important to reiterate, however, that overall, dogs had primarily mildly fearful reactions to the cone and that behaviors suggesting extreme fear or aggression were rarely recorded. Both of the other stimuli (the stranger and the dog statue), although less apparently intimidating, still provoked a certain amount of fear in a moderate portion of the study population. These findings, overall, are encouraging, although they also highlight a continued need for improvement. What constitutes a minimum to optimal amount of socialization in dogs has yet to be determined [31,32]. However, introducing stimuli in a positive and gradual way, especially during the early stages of life, has been reported to help minimize the development of fear-related behaviors [33,34]. Hence, it is advisable that breeders have socialization protocols in place that incorporate controlled exposure to both social and non-social stimuli. Such protocols should ultimately aim to improve the dogs’ welfare and to maximize chances of success after retirement and rehoming. Indeed, further research is needed to fully understand which management practices and/or protocols may be more effective for evidence-based welfare improvements in this population of dogs. Simple behavioral tests like the one presented here can be used to explore the effects of short, targeted interventions where, for example, adult dogs are re-tested after simple socialization practices have been implemented.

## Figures and Tables

**Table 1:** Study population demographics: a list of breeds, number of dogs and sex per breed is provided.

Breed	Female	Male	Total
American Cocker Spaniel	4	1	5
Australian Shepherd	3	0	3
Bernese Mountain Dog	1	0	1
Bichon Frise	3	0	3
Boston Terrier	1	1	2
Boxer	1	0	1
Bullmastiff	2	0	2
Cavalier King Charles Spaniel	3	2	5
French Bulldog	2	0	2
Golden Retriever	0	1	1
Great Dane	1	1	2
Havanese	4	2	6
Lhasa Apso	2	0	2
Maltese	1	1	2
Miniature Schnauzer	3	0	3
Neopolitan Mastiff	2	0	2
Pomeranian	3	1	4
Saint Bernard	1	0	1
Samoyed	2	0	2
Shiba Inu	1	0	1
Shih Tzu	2	0	2
Siberian Husky	0	1	1
Standard Poodle	1	0	1
Toy Poodle	0	2	2
Yorkshire Terrier	2	2	4
**Grand Total**	**45**	**15**	**60**

**Table 2:** Ethogram used to analyze the behavior of dogs. Behaviors were scored in terms of duration or frequency of occurrence.

Category	Behavior	Definition	Type of Score
Fear	Escaping ^†^	Moving to back or front exit, pawing at door or ground near door	Duration
	Freezing ^†^	Dog stops movement or sound, body still, muscle tension, possibly in a lowered or stiff/rigid posture	Duration
	Retreat ^†^	Dog moves away from the stimulus with lowered posture (possibly tail tucked, ears back, body lowered to ground) after investigation	Duration
	Gaze avoidance	Dog continually looks at and looks away from stimulus	Duration
Stress	Yawning ^†^	Opening mouth wide, no noise generated, could be repetitive	Frequency
	Paw-lifting	Raising paw off the ground and maintaining for a short period of time	Frequency
	Shivering ^†^	Entire body is shaking; could be in a lowered posture (possibly tail tucked, ears back)	Duration
	Lip-licking	Tongue repeatedly moving around the outside of the mouth	Frequency
	Body shake ^†^	Rapid shake of body from head to tail	Frequency
Aggression	Biting/Snapping ^†^	Opening mouth and showing teeth and quickly closing, could be snapping at the air	Frequency
	Teeth baring ^†^	Lips raised showing teeth, could include growling or snarling	Frequency
Stereotypic	Pacing ^†^	Dog walking from side to side of kennel in a repetitive manner (at least 3 times)	Duration
	Circling ^†^	Dog moving in circles around themselves or an object in a repetitive manner (at least 3 times)	Duration
Activity	Standing ^1^	Dog is on all four legs, not moving	Duration
	Sniffing ^1^	nose on or toward stimulus, mouth closed, breathing rapidly	Duration
	Lying	Dog is flat on the ground, head can be up or also on the ground, back and tail in neutral position for breed	Duration
	Walking	Dog moving around pen with neutral back and tail positions for breed	Duration
	Sitting ^1^	Dog back legs are tucked under body, front legs vertical	Duration
	Standing on hind legs	Dog is standing on back legs with front legs in the air or leaning against the wall	Duration
Non-fearful	Stimulus directed play	Any of the following in combination: tail wagging, ears perked, sniffing/licking/pawing stimulus, moving stimulus, play mouthing, play bow	Duration
	Affiliative approach to tester	Dog moves toward tester with a loose body posture, tail high or low, tail possibly wagging, ears possibly perked, possibly sniffing tester or air near tester	Duration
Vocalization	Bark	Quick and possibly repetitive vocalizations	Frequency
	Whine ^†^	Higher-pitched vocalization	Frequency
	Growl ^†^	Lower-pitched grumble vocalization, may have teeth showing	Frequency
Other	Investigating environment	Sniffing/licking the floor or pen walls (not directed toward stimulus)	Duration
	Elimination ^†^	Dog urinates, defecates, or vomits on the pen floor	Frequency
	Tester contact/No contact	Tester was able/unable to make contact with the dog during the approach test	Frequency
	Takes treat	Dog accepts treat from tester or from the floor	Frequency
	Latency to approach and interact with stimulus	Time elapsed from the start of the test to the dog approaching either the tester or the object	Duration
Posture Modifiers	Lowered	head lowered, ears pinned back, center of body lowered, tail tucked	N/A
	Neutral	head normal to raised, ears forward, tail high or normal for breed	
	Rigid	head high, ears forward, tail high, mouth tight, muscles tensed	

^†^ Behaviors recorded but not included in analysis due to very low occurrence (see results); ^1^ Posture modifiers (i.e., attributes of behaviors) were applied.

**Table 3:** Intraclass Correlations Coefficient (ICC) for analyzed behaviors. Values listed are ICC values and the lower and upper bounds of the 95% confidence interval (CI).

Behavior	ICC	95% Confidence Interval	
		Lower Bound	Upper Bound
Gaze avoidance	0.98	0.95	0.99
Investigating environment	0.97	0.93	0.99
Stimulus directed play	0.98	0.95	0.99
Sitting	0.98	0.96	0.99
Sniffing	0.98	0.96	0.99
Standing	0.72	0.39	0.87
Walking	0.77	0.49	0.89
Lowered posture	0.82	0.60	0.92
Neutral posture	0.79	0.53	0.90
Rigid posture	0.87	0.71	0.94
Lying	0.89	0.76	0.95
Standing on hind legs	0.99	0.99	0.99
Affiliative approach to tester	0.74	0.43	0.88
Latency to approach and interact	0.95	0.90	0.98
Takes treat	0.96	0.90	0.98
Tester contact/No contact	1.000	1.00	1.00
Stress-related	0.98	0.95	0.99

**Table 4:** Cross tabulation analysis for red, yellow, and green categories (based on [10]) for key behaviors. Values in the table represent the average duration in seconds spent in the listed behavior when subjects were exposed to the cone stimulus.

Behavior	Red	Yellow	Green
Gaze avoidance	4.67	3.62	1.35
Investigate environment	4.59	3.63	6.81
Stimulus directed play	0	0	0.11
Lying	0.85	0.39	0.44
Standing on hind legs	0	0	0.77
Retreat	0.75	1.16	0.38
Sitting	9.75	5.66	4.85
Sniffing	1.68	2.41	4.85
Standing	10.99	8.94	7.29
Walking	4.66	7.10	12.16
Latency to approach and interact with the stimulus	20.74	16.03	6.75
Lowered posture	15.38	9.47	5.36
Neutral posture	3.29	2.30	8.78
Rigid posture	3.75	5.25	1.92
Stress-related	0.23	0.36	0.28

**Table 5:** Cross tabulation analysis for red, yellow, and green categories (based on [10]) for significant behaviors. Values in the table represent the average duration in seconds spent in the listed behavior when subjects were exposed to the dog statue stimulus.

Behavior	Red	Yellow	Green
Gaze avoidance	2.66	1.57	1.44
Investigate environment	0.58	1.05	1.24
Stimulus directed play	0	0	0.69
Lying	0	0.09	0.22
Standing on hind legs	0	0.21	0.21
Retreat	0.89	0.82	0.95
Sitting	2.50	0	1.38
Sniffing	15.95	17.72	16.67
Standing	6.21	5.97	4.39
Walking	8.72	9.12	5.01
Latency to approach and interact with the stimulus	5.64	1.06	1.74
Lowered posture	15.52	15.45	7.16
Neutral posture	0.54	1.77	7.33
Rigid posture	8.60	6.46	7.95
Stress-related	0.54	0.36	0.61

## Data Availability

Data will be provided in an online repository after acceptance of the manuscript.
